# An Unlabeled Electrochemical Immunosensor Uses Poly(thionine) and Graphene Quantum Dot-Modified Activated Marigold Flower Carbon for Early Prostate Cancer Detection

**DOI:** 10.3390/bios14120589

**Published:** 2024-12-02

**Authors:** Suparat Cotchim, Supatinee Kongkaew, Panote Thavarungkul, Proespichaya Kanatharana, Warakorn Limbut

**Affiliations:** 1 Center of Excellence for Trace Analysis and Biosensor, Prince of Songkla University, Hat Yai 90110, Thailand; cs.suparat@gmail.com (S.C.); supatinee_fang@hotmail.com (S.K.); panote.t@psu.ac.th (P.T.); proespichaya.k@psu.ac.th (P.K.); 2 Center of Excellence for Innovation in Chemistry, Faculty of Science, Prince of Songkla University, Hat Yai 90110, Thailand; 3 Division of Health and Applied Sciences, Faculty of Science, Prince of Songkla University, Hat Yai 90110, Thailand; 4 Division of Physical Science, Faculty of Science, Prince of Songkla University, Hat Yai 90110, Thailand; 5 Forensic Science Innovation and Service Center, Prince of Songkla University, Hat Yai 90110, Thailand

**Keywords:** marigold flowers, activated carbon, graphene quantum dots, poly(thionine), prostate cancer

## Abstract

The activated carbon from marigold flowers (MG) was used to make an unlabeled electrochemical immunosensor to determine prostate cancer. MG was synthesized by hydrothermal carbonization and pyrolysis. MG had a large surface area, was highly conductive, and biocompatible. MG modified with graphene quantum dots produced excellent electron transfer for grafting poly(thionine) (PTH). The amine group of PTH bonded with anti-prostate-specific antigen (Anti-PSA) via glutaraldehyde, forming a layer that improved electron transfer. The binding affinity of the immunosensor, presented as the dissociation constant (Kd), was calculated using the Langmuir isotherm model. The results showed that a lower Kd value indicated greater antibody affinity. The immunosensor exhibited two different linear ranges under optimized conditions: 0.0125 to 1.0 ng mL^−1^ and 1.0 to 80.0 ng mL^−1^. The sensor could detect concentrations as low as 0.005 ng mL^−1^, and had a quantification limit of 0.017 ng mL^−1^. This immunosensor accurately quantified PSA levels of human serum, and the results were validated using enzyme-linked fluorescence assay (ELFA).

## 1. Introduction

Cancer of the prostate, also known as prostate cancer (PCa), is the most common form of cancer in men and ranks as the fourth most common form of cancer diagnosed worldwide [1]. Among men of middle age and older, prostate cancer is considered to be the “invisible killer” [2]. People aged 65 and older are diagnosed with approximately sixty percent of all cases of prostate cancer. In the initial phases of the disease, none of the symptoms associated with prostate cancer are readily apparent. However, when the cancer is discovered in its later stages, the rate of death is significantly higher than it is in earlier stages.

A determination of the serum biomarker prostate-specific antigen (PSA), which is a glycoprotein that is secreted by prostate epithelial cells, is required in order to diagnose patients with prostate cancer [3]. PSA levels in healthy individuals are typically below 4.0 ng mL^−1^. For this reason, the threshold value for the clinical diagnosis of PCa is 4.0 ng mL^−1^, respectively. A serum PSA level exceeding 10 ng mL^−1^ is regarded as a high risk for PCa. However, because the percentage of accurate PCa diagnoses ranges between 70 and 80 percent [4,5], early diagnostic methods that are more accurate and reliable have the potential to improve survival rates for PCa. It is common practice to use a digital rectal exam (DRE) as the initial screening test for PCa. However, it is possible that DRE does not significantly reduce death rates and can result in a large number of false positives, which can lead to unnecessary invasive diagnostic procedures [6]. PSA biomarker assays that emphasize the specific affinity binding between PSA antigens and antibodies offer greater precision.

In recent years, PSA has been detected by a variety of newly developed assays, which have included an enzyme-linked immunosorbent assay (ELISA) [7], fluorescent immunoassays [8], a radioimmunoassay [9], and electrochemical immunosensors [3,10]. Electrochemical immunosensors are favored due to their exceptional sensitivity, portability, cost-effectiveness, and simple fabrication [2,11]. Nanomaterials are extensively employed to enhance electrode surfaces in electrochemical immunosensors, thereby increasing antibody loading efficiency. Materials such as metal nanoparticles [12,13], carbon nanotubes [14,15], graphene [16,17], and activated carbon [18] can enhance the sensitivity, reproducibility, and selectivity of electrochemical immunosensors.

Activated carbon is both porous and cost-effective. Furthermore, it exhibits catalytic properties, a substantial specific surface area, and exceptional chemical stability [19]. Precursors for activated carbon production include various types of flowers. In Thailand, marigold flowers (MG) are discarded after religious ceremonies [20]. This study focuses on the synthesis of activated carbon from MG using hydrothermal carbonization (HTC) and pyrolysis processes. Another interesting nanomaterial at the moment is quantum dots (QDs), which are useful for electrode modification due to their high surface area-to-volume ratio, making them suitable for use in the development of medical diagnostic sensors [21,22,23]. However, QDs are often decorated with metal nanoparticles and can release toxic heavy metals. Graphene quantum dots (GQDs) are nanoscale graphene particles that combine properties of both graphene and carbon dots [24]. Importantly, GQDs exhibit biocompatibility. They provide two additional advantages: signal amplification and electro-catalytic activity. They produce a substantial quantity of active sites [25,26,27] due to their exceedingly small particle size, generally ranging from 1 to 10 nm [28], in addition to their extensive specific surface area. GQDs also offer excellent electron donor and acceptor properties [29,30], which are beneficial for electrochemical sensors [31]. Electrochemical immunosensors require an immobilized antibody, and thionine (TH) is particularly well suited for this purpose due to its excellent chemical stability and electroactivity [32]. The purpose of this study is to increase the biocompatibility of an immunosensor that can detect PSA for early PCa diagnosis. To achieve this, we combine TH with GQDs and activated carbon.

## 2. Materials and Methods

### 2.1. Reagents and Materials

Prostate-specific antigen (PSA), mouse monoclonal anti-PSA, cancer antigen 153 (CA153), cancer antigen 19-9 (CA19-9), carcinoembryonic antigen (CEA), and cancer antigen 125 (CA125) were purchased from Fitzgerald Industries International (Acton, MA, USA). Human serum albumin (HSA) was purchased from Dako (Glostrup, Denmark). Bovine serum albumin (BSA), glucose (Glu), uric acid (UA), ascorbic acid (AA), potassium ferricyanide, potassium ferrocyanide, potassium chloride, thionine acetate, and glutaraldehyde were from Sigma-Aldrich (Steinheim, Germany). Dipotassium hydrogen o-phosphate and potassium dihydrogen o-phosphate were from Ajax Finechem (Sydney, Australia). Samples of clinical serum were obtained from Hat Yai Hospital, which is located in Hat Yai, Thailand. Ultrapure water (18.2 MΩ·cm) was prepared for all solution preparation using the Barnstead Easy Pure II system (Thermo Scientific, Waltham, MA, USA). Screen-printed electrodes (SPE) with a 3 mm diameter working electrode were sourced from PalmSens (Utrecht, The Netherlands).

### 2.2. Instruments

FE-SEM (field emission scanning electron microscopy, Apreo, FEI, Eindhoven, The Netherlands) was used to investigate the morphology of the MG. FE-TEM (field emission transmission electron microscopy, Talos F200i, Thermo Scientific, Brno, Czech Republic) and UV-VIS spectrophotometry (T60UV, PG Instruments Limited, Lutterworth, UK) were employed to examine the morphology of the GQDs. A PalmSens4 potentiostat-galvanostat (PalmSens BV, Utrecht, The Netherlands) was used for electrochemical analyses such as CV (cyclic voltammetry), EIS (electrochemical impedance spectroscopy), and DPV (differential pulse voltammetry).

### 2.3. Fabrication of Immunosensor

#### 2.3.1. Synthesis of Activated Carbon from MG

Marigold flowers were obtained from the Sri-Trang market, Songkhla, Thailand, cleaned with ultrapure water, and desiccated at 80 °C for 24 h in a hot-air oven. The desiccated flowers were severed into diminutive fragments and pulverized into a fine powder utilizing a mortar. Ten grams of the powder were combined with 50.0 mL of ultrapure water. The resultant mixture was subsequently positioned in a Teflon-lined stainless-steel autoclave and subjected to heating for 24 h at 180 °C.

The dried material was mixed with KOH in a 1:1 weight ratio and heated at a rate of 10 °C per minute until reaching a temperature of 800 °C, with nitrogen gas flowing at a rate of 0.2 L per minute [20]. Pyrolysis persisted for one hour at 800 °C. The product was treated with an 11.0 mol L^−1^ H_2_SO_4_ solution and ultrapure water to eliminate residual KOH, then dried for 24 h at 80 °C to yield activated carbon from marigold flower (MG).

#### 2.3.2. Synthesis of GQDs

GQDs were synthesized through the hydrothermal treatment of graphene oxide (GO) with ammonia [33]. A suspended GO (5.0 mg mL^−1^) was prepared in ultrapure water, and 5.0 mL of this solution was combined in a glass vial with 3.0 mL of 28% ammonia solution and 5.0 mL of ultrapure water. The mixture was stirred for 30 min before being heated for 5 h at 90 °C in a Teflon-lined autoclave. A yellow supernatant containing GQDs was obtained by filtering the insoluble precipitate with a 0.22 µm nylon syringe filter after it had cooled to room temperature. The supernatant was heated to 100 °C for one hour to evaporate excess ammonia. Upon reaching room temperature, the supernatant was subjected to filtration for 2 days using a dialysis membrane with a molecular weight cut-off of 2000 Da.

#### 2.3.3. Immunosensor Fabrication

**Figure 1:**
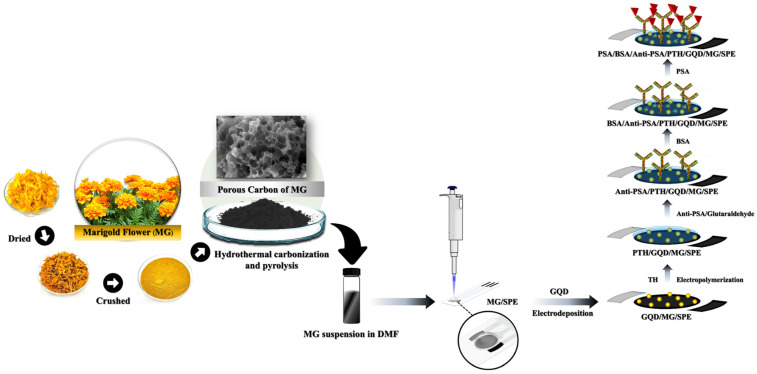
The diagram illustrates the various stages involved in the fabrication process of the proposed label-free immunoelectrode.

An SPE was modified by drop-casting 2.0 µL of 4.0 mg mL^−1^ MG onto the working electrode surface (MG/SPE). The MG/SPE was dried at room temperature. Subsequently, GQDs (2.0 mg mL^−1^) were electrodeposited on the MG/SPE by cyclic voltammetry, scanning from 0 to −1.4 V at 0.05 V s^−1^ for a total of ten cycles to produce a GDQ/MG/SPE. TH was then electropolymerized on the surface of the GQD/MG/SPE, using CV. Electropolymerization was conducted in a solution of 0.25 mmol L^−1^ TH monomer in 0.1 mol L^−1^ phosphate buffer (PB, pH 7.4), scanning from −0.7 to 1.2 V at a total of 0.05 V s^−1^ for 15 cycles to produce the PTH/GQD/MG/SPE. The PTH/GQD/MG/SPE was then treated with 2.5% glutaraldehyde for 20 min to immobilize Anti-PSA. The antibody was solubilized in PB at a concentration of 40.0 g mL^−1^, and 1.5 µL of the solution was applied to the electrode surface and incubated for 12 h at 4 °C. During incubation, amine groups in the PTH film were covalently bound to Anti-PSA by glutaraldehyde to produce an Anti-PSA/PTH/GQD/MG/SPE. The Anti-PSA/PTH/GQD/MG/SPE was rinsed with PB and incubated in a 1.0% BSA solution for 60 min to inhibit non-specific binding on the electrode surface. The BSA/Anti-PSA/PTH/GQD/MG/SPE was preserved at 4 °C prior to utilization. The fabrication procedure of the proposed label-free immunoelectrode was shown in Figure 1. The electrochemical properties of the electrode during each preparation process were examined through CV and EIS.

### 2.4. Measurements of Label-Free Electrochemical Immunosensors

DPV was used to conduct a quantitative study of the PSA biomarker for PCa detection. A change in the signal level at [Fe(CN)_6_]^3−/4−^ (5.0 mmol L^−1^) indicated the formation of the antigen-antibody immunocomplex. The PSA concentration in the sample was determined by the change in current response (ΔI) using: ΔI = I_initial_ − I_final_, where I_initial_ is the current before and I_final_ after the antigen-antibody immunocomplex formation.

### 2.5. Real Samples Preparation

The developed immunosensor was employed to measure PSA levels in human serum samples from both patients and healthy individuals. The samples were obtained from Hat Yai Hospital, with ethical approval from the hospital’s committee. A solution of 0.1 mol L^−1^ PB with a pH of 7.4 was used to dilute the samples. Then, 2.0 µL of diluted serum sample were dropped onto the immunoelectrode and the DPV response was analyzed. It was assessed whether the results from the developed immunosensor were comparable to those obtained using enzyme-linked fluorescence assay (ELFA). There was a comparison of the data obtained from both methods using a paired *t*-test.

## 3. Results and Discussion

### 3.1. Morphological Characterization

**Figure 2:**
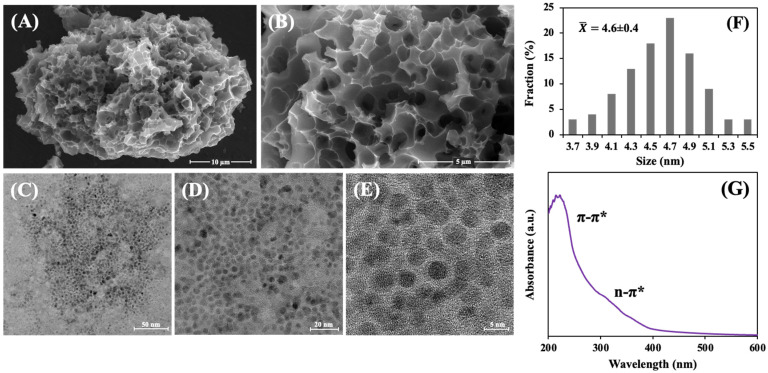
FE-SEM images of MG activated carbon at 5000× (**A**) and 15,000× (**B**) magnifications. FE-TEM images at different magnifications are of GQDs prepared via hydrothermal treatment in NH_3_ (**C**–**E**). Diameter distributions of GQD (**F**) and UV-vis spectrum of GQD (**G**).

MG morphology was characterized by FE-SEM, as shown in Figure 2A,B. The morphology of MG was sponge-like, with a continuous pore network on the surface, indicating the significant effect of hydrothermal carbonization, KOH activation, and pyrolysis. The activation mechanism of the reaction using KOH can be described by three main mechanisms (chemical activation, physical activation, and intercalation) [34,35,36]: (1) Chemical activation is responsible for generating a pore structure through the formation of metallic K during activation with KOH, as shown in reactions (1). Additionally, in reactions (4) and (5), K_2_CO_3_ and K_2_O react with carbon to produce metallic K and CO. These reactions enhance pore formation by removing carbon atoms and forming additional reactive sites on the surface of the material. The formation of metallic K in these reactions also contributes to the expansion and restructuring of the carbon framework, leading to increased microporosity and enhanced surface area. (2) Physical activation involves the gasification of carbon, as shown in reactions (2). This CO_2_ subsequently reacts with carbon to form CO in reaction (3). These reactions contribute to the development of mesopores by removing carbon atoms, enhancing the pore structure, and increasing the surface area of the material. (3) Intercalation occurs as metallic K interacts with the carbon matrix, as shown in reactions (1), (4), and (5). During this process, metallic K is intercalated into the carbon framework, contributing to pore formation and structural reorganization, which enhance the surface area of the material and electrochemical properties. These combined processes demonstrate how chemical activation, physical activation, and intercalation work synergistically to create the porous structure of MG, significantly enhancing its effectiveness in applications such as electrochemical sensing.

6KOH + 2C → 2K + 3H_2_ +2K_2_CO_3_ (1)

K_2_CO_3_ → K_2_O + CO_2_ (2)

CO_2_ + C → 2CO (3)

K_2_CO_3_ + 2C → 2K + 3CO (4)

C + K_2_O → 2K + CO (5)

Figure 2C–E displays typical FE-TEM images of GQDs, while Figure 2F presents the diameter distributions of GQDs. Within the range of 3.7 to 5.5 nm, the GQDs exhibited uniform diameters, with an average diameter of 4.6 ± 0.4 nm. Figure 2G displays the UV–vis spectrum of the GQDs. A typical absorption peak was observed, and it contained both the π-π* and the n-π* structures. As a result of the aromatic sp^2^ hybridized carbon atoms, the π-π* bonds were attributed to the C=C bonds, while the n-π* bonds were attributed to the oxygen present in the GQDs [37,38].

The sponge-like structure of MG could provide an excellent matrix for further functionalization, and the synthesized GQDs exhibited uniform size distribution and specific optical properties, making them suitable for use in electrochemical sensor applications. These results showed that MG and GQDs could be suitable materials for a high-performance electrochemical immunosensor.

### 3.2. Characterization of the Electrical Activities of Materials

**Figure 3:**
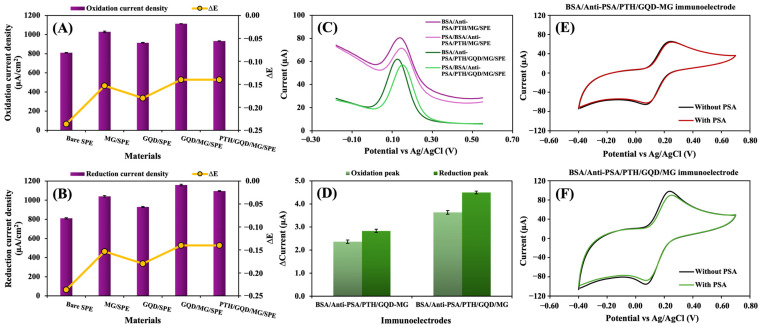
The bar chart shows the oxidation current density (**A**) and reduction current density (**B**) for a bare SPE, MG/SPE, GQD/SPE, GQD/MG/SPE, and PTH/GQD/MG/SPE. The DPV response curve at the fabricated immunoelectrode with and without GQDs (**C**). The bar chart shows the PSA binding at immunoelectrodes fabricated by two different methods: one in which mixed GQDs and MG (GQD-MG) were electrodeposited on the SPE and another in which the SPE was modified by drop casting MG, followed by the electrodeposition of GQDs on the MG/SPE (**D**). The voltammograms associated with (**D**) were produced in 5.0 mmol L^−1^ [Fe(CN)_6_]^3−/4−^ that contained 0.10 mol L^−1^ KCl, either with or without PSA at 1.0 ng mL^−1^ (**E**,**F**). The BSA/Anti-PSA/PTH/GQD-MG/SPE and BSA/Anti-PSA/PTH/GQD/MG/SPE were fabricated with 8.0 µg of MG, GQDs electrodeposited for 10 cycles, and 1.0 mmol L^−1^ TH-monomer.

The CV was used to characterize the electrical behaviors of the bare SPE, MG/SPE, GQD/SPE, GQD/MG/SPE, and PTH/GQD/MG/SPE in a solution of 5.0 mmol L^−1^ [Fe(CN)_6_]^3−/4−^ with 0.10 mol L^−1^ KCl. Figure 3A,B display the results of calculations that were performed on the oxidation and reduction current densities that were produced at each of the materials. Due to the synergistic effect of MG and GQDs, the GQD/MG/SPE showed the highest oxidation and reduction current densities. The large pore volume of MG, which boosts adsorption capacity, and the GQDs, which offer a conductive substrate for electron transfer via [Fe(CN)_6_]^3−/4−^, contributed to an increase in the electrochemical active surface area. The GQD/MG/SPE showed the lowest ∆E value, indicating increased electrode surface and the rate of electron transfer. The presence of the PTH film on the electrode surface lowered both the oxidation and reduction current densities in PTH/GQD/MG/SPE.

The affinity of PSA to the fabricated electrode was investigated with and without GQDs to confirm the benefit of modifying MG with GQDs. The DPV responses with GQDs (green line) and without GQDs (purple line) are shown in Figure 3C. GQDs helped to reduce the background current, providing a higher current response than was obtained without GQDs. These measurements validated the efficiency of modifying the surface of MG with GQDs, a significant finding in our research. Moreover, two methods of modifying MG were tested. GQDs and MG particles were mixed (GQD-MG), and the mixture was electrodeposited on the SPE, and MG was drop-cast on the SPE, followed by the electrodeposition of GQDs (GQD/MG). The layer-by-layer modification of the GQD/MG electrode exhibited higher oxidation and reduction peak currents (Figure 3D) compared to the mixed GQD-MG modification. This is attributed to the increased active surface area achieved through the layer-by-layer approach. These novel preparation methods, tested for the first time in this research, provide exciting insights into the electrochemical properties of the materials. The voltammograms shown in Figure 3E,F further highlighted the findings of this study. These electrochemical characterizations confirmed that modifying the SPE with GQD/MG significantly enhanced electrode performances due to the synergetic effects of MG and GQDs. The layer-by-layer assembly method proved superior to the mixed particle method and improved electrochemical activity. These results confirmed the suitability of modification with GQDs and provided new information about the fabrication of electrochemical immunosensors.

### 3.3. Electrochemical Characterization

When analyzing surface modification of the immunosensor, CV and EIS were utilized. The reaction mixture consisted of 5.0 mmol L^−1^ [Fe(CN_6_)]^3−/4−^ and 0.10 mol L^−1^ KCl. The electrodes characterized were the bare SPE, MG/SPE, GQD/MG/SPE, PTH/GQD/MG/SPE, Anti-PSA/PTH/GQD/MG/SPE, BSA/Anti-PSA/PTH/GQD/MG/SPE, and PSA/BSA/Anti-PSA/PTH/GQD/MG/SPE. Figure S1A in the electronic Supplement Materials shows a well-defined redox peak of [Fe(CN_6_)]^3−/4−^ at the bare SPE (black line). When MG was deposited on the surface of the electrode (orange line), the redox peak current was higher because of the increased electrode surface and electron transfer rate. The electrodeposition of GQDs on the MG/SPE (purple line) enhanced the conductance value at a lower ∆E, indicating that GQDs improved kinetic charge transfer with the redox probe. After PTH was formed (blue line), the redox peak current decreased because of the PTH film layer on the electrode surface. When Anti-PSA was immobilized on the PTH surface (green line), the amine groups of the PTH structure could interact with the Anti-PSA, and the redox peak current decreased further. After Anti-PSA was immobilized with BSA (dark blue line), the redox peak current of [Fe(CN_6_)]^3−/4−^ decreased again. When the immunoelectrode detected PSA (red line), the redox peak current decreased because PSA bound to Anti-PSA, hindering electron transfer at the immunoelectrode.

In a solution of 5.0 mmol L^−1^ [Fe(CN)_6_]^3−/4−^ with 0.10 mol L^−1^ KCl, electrochemical impedance spectroscopy (EIS) was utilized to investigate the interfacial properties and changes in electron transfer resistance. With a sampling rate of ten points per second, the frequency range was from 100 kHz to 0.05 Hz. At a magnitude of the applied potential was 0.01 V. As shown in Figure S1B, the Nyquist plot for the bare SPE (black line) presented a large semicircle, indicating a charge transfer resistance (R_ct_) = 9919 ± 1 Ω. The EIS spectra of the MG/SPE (orange line) and GQD/MG/SPE (purple line) formed straight lines, indicating the high conductivity of the materials. The semicircle diameter formed by the PTH/GQD/MG/SPE (blue line) indicated an R_ct_ = 1241 ± 0.5 Ω. The decrease in charge transfer resistance was due to the PTH polymer film that reduced the rate of electron transfer between the surface of the electrode and [Fe(CN)_6_]^3−/4−^ solution. The plots of the Anti-PSA/PTH/GQD/MG/SPE (green line) and BSA/Anti-PSA/PTH/GQD/MG/SPE (dark blue line) presented semicircles that indicated R_ct_ values = 3289 ± 0.7 Ω and 4809 ± 1 Ω, respectively, confirming that Anti-PSA and BSA were successfully immobilized on the electrode surface. When PSA was detected (red line), the semicircle diameter indicated an R_ct_ = 5774 ± 1 Ω. This result confirmed the insulating property of the antigen-antibody immunocomplex.

These CV and EIS studies provided evidence that the proposed immunosensor’s stepwise fabrication results in a successful product. The significant changes in redox peak currents and charge transfer resistance at each modification step confirmed the effective immobilization of MG, GQDs, PTH, Anti-PSA, and BSA. The enhanced electrical activities observed after modification with GQD/MG highlighted the synergetic effects of these materials, leading to improved electrode performance. The final immunosensor showed a clear response to PSA, validating its potential application for sensitive and specific PCa biomarker detection.

### 3.4. The Specificity of PSA Detection

To confirm the efficiency of the proposed immunosensor, the responses of the immunoelectrode were evaluated with and without Anti-PSA, both in the presence and absence of PSA. The BSA/PTH/GQD/MG/SPE and the BSA/Anti-PSA/PTH/GQD/MG/SPE were first tested in a solution of 5.0 mmol L^−1^ [Fe(CN)_6_]^3−/4−^ with 0.10 mol L^−1^ KCl, containing 1.0 ng mL^−1^ of PSA. The ΔI results indicated no significant difference (*p* > 0.05) when PSA was not present (Figure S2). The response of the proposed immunoelectrode was significant only when both Anti-PSA and PSA were present, confirming the specificity of the immunosensor and ensuring reliable PSA detection for PCa diagnosis.

### 3.5. Optimizations

#### 3.5.1. The Effect of MG Loading

To optimize the amount of MG used to fabricate the immunosensor, the immunoelectrode was fabricated with different amounts of MG, ranging from 0 μg to 16.0 μg. The oxidation and reduction current density of each condition was determined by employing CV in a solution of 5.0 mmol L^−1^ [Fe(CN)_6_]^3−/4−^ with 0.10 mol L^−1^ KCl. When the amount of MG used was increased from 2.0 to 8.0 μg, the oxidation and reduction current densities of the immunosensor gradually increased (Figure S3A,B) because of the larger surface area, enhanced pore edge activity, and superior conductivity. Nevertheless, with a further increase in the amount of MG, the oxidation and reduction current density of [Fe(CN)_6_]^3−/4−^ reached a plateau, indicating that the active sites on the electrode surface had been saturated. Therefore, 8.0 μg of MG was identified as the most suitable amount for the subsequent studies.

#### 3.5.2. The Effect of GQD Loading

The number of GQDs electrodeposition cycles was varied from 0 to 15 cycles using CV in a solution of 5.0 mmol L^−1^ [Fe(CN)_6_]^3−/4−^ with 0.10 mol L^−1^ KCl. The oxidation and reduction current density of [Fe(CN)_6_]^3−/4−^ increased from 0 to 10 cycles due to the better electroactivity of the GQDs. After 10 cycles, the oxidation and reduction current densities of [Fe(CN)_6_]^3−/4−^ reached a plateau (Figure S3C,D). Therefore, 10 electrodeposition cycles were determined to be the most suitable parameter for the following studies.

#### 3.5.3. The Effect of TH Concentration

**Figure 4:**
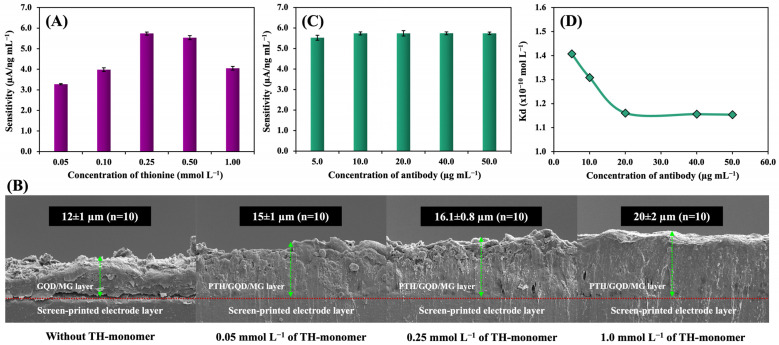
The effect on electrode sensitivity of thionine concentration (**A**). The cross-sectional views of the PTH/GQD/MG/SPE show the correlation between PTH film thickness and TH-monomer concentration (**B**). The chart (**C**) shows the effect of antibody concentration on electrode sensitivity. (**D**) The Kd value of the antigen-antibody immunocomplex at different antibody concentrations was determined by using DPV in 5.0 mmol L^−1^ [Fe(CN)_6_]^3−/4−^ solution with 0.10 mol L^−1^ KCl.

The effect of TH-monomer concentration was studied within the range of 0.05 to 1.00 mmol L^−1^, while PSA concentrations varied from 0.2 to 1.0 ng mL^−1^. DPV was used to detect increments of PSA in a solution of 5.0 mmol L^−1^ [Fe(CN)_6_]^3−/4−^ with 0.10 mol L^−1^ KCl. The best PTH film growth was obtained at a TH-monomer concentration of 0.25 mmol L^−1^ (Figure 4A). When PTH was formed, the amine groups of the structure could interact with the Anti-PSA. TH-monomer concentrations > 0.25 mmol L^−1^ produced a thick film with higher nucleation of PTH and reduced the sensitivity of the immunosensor slightly. To confirm the increasing thickness of the polymer film, cross-sectional SEM images of electrodes fabricated at TH-monomer concentrations of 0, 0.05, 0.25, and 1.0 mmol L^−1^ (*n* = 10) were analyzed (Figure 4B). The thicknesses of the polymer films were found to be 12 ± 1, 15 ± 1, 16.1 ± 0.8, and 20 ± 2 µm, respectively, confirming that the PTH film thickness on the electrode surface increased as the concentration of the TH-monomer increased. Consequently, 0.25 mmol L^−1^ of TH monomer was selected as the most suitable condition for subsequent studies.

#### 3.5.4. The Effect of Antibody Concentration

The influence of varying concentrations on the immunosensor’s performance was examined by incubating the immunoelectrode with Anti-PSA in concentrations ranging from 5.0 to 50.0 μg mL^−1^ at 4 °C for 12 h. The antibody was subsequently immobilized by incubating it with 1.0% BSA for 60 min. The immunoelectrode was washed with PB and used to detect PSA at a range of concentrations, including 0.2, 0.4, 0.6, 0.8, and 1.0 ng mL^−1^, using DPV in a solution of 5.0 mmol L^−1^ [Fe(CN)_6_]^3−/4−^ with 0.10 mol L^−1^ KCl (Figure 4C). The sensitivity of the immunosensor increased as the antibody concentration increased from 5.0 to 40.0 μg mL^−1^, then reached a plateau after further increments of Anti-PSA concentration. The saturation of binding between Anti-PSA and the surface of the immunoelectrode described the result obtained.

The antigen-antibody dissociation constants (Kd) of the immunoelectrode were calculated using the Langmuir isotherm model as follows: IF = Imax[F]/KD + [F], where Imax represents the maximum current response when antigen binds to the antibody, F represents the PSA concentration, IF represents the current response, and Kd represents the dissociation constant. The Kd value of the antigen-antibody immunocomplex was derived from the linear regions of the corresponding 1/IF versus 1/[F] plots (Figure 4D). For antibody concentrations of 5.0, 10.0, 20.0, 40.0, and 50.0 μg mL^−1^, the Kd values were 1.407 ± 0.006 × 10^−10^ mol L^−1^, 1.309 ± 0.004 × 10^−10^ mol L^−1^, 1.161 ± 0.001 × 10^−10^ mol L^−1^, 1.156 ± 0.001 × 10^−10^ mol L^−1^, and 1.154 ± 0.001 × 10^−10^ mol L^−1^, respectively. According to the immobilization results, the optimal antibody concentration was determined to be 40 μg mL^−1^.

#### 3.5.5. The Effect of Incubation Time on Immunocomplex Formation

**Figure 5:**
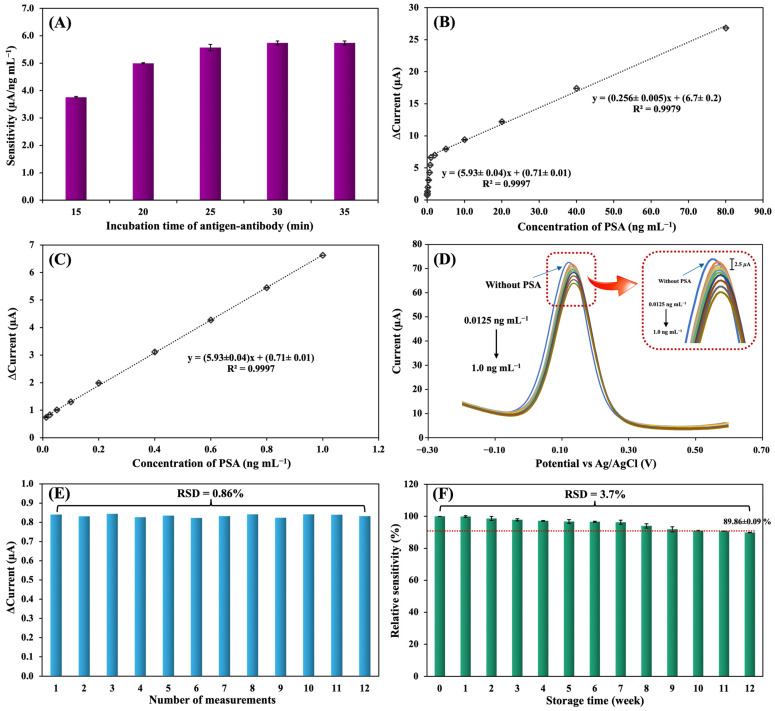
The effect on electrode sensitivity of antigen-antibody incubation time (**A**). Calibration curves of the current change (ΔI) and PSA concentration in the range of 0.0125 to 80.0 ng mL^−1^ were generated from DPV measurements in a solution of 5.0 mmol L^−1^ [Fe(CN)_6_]^3−/4−^ with 0.10 mol L^−1^ KCl (**B**). Calibration curves of the current change (ΔI) and PSA concentration in the range of 0.0125 to 1.0 ng mL^−1^ (**C**). DPV voltammogram for different PSA concentrations obtained from 0.0125 to 1.0 ng mL^−1^ (**D**). The operational lifetime of one immunoelectrode preparation (**E**). The storage stability of the immunoelectrode (**F**). DPV conditions: potential range of −0.2 to +0.6 V (vs. Pseudo-Ag/AgCl), scan rate of 50 mV s^−1^, E pulse of 75 mV, t pulse of 200 ms, and E step of 5.0 mV.

The influence of antigen-antibody incubation time was studied at intervals of 15, 20, 25, 30, and 35 min for detecting PSA levels of 0.2, 0.4, 0.6, 0.8, and 1.0 ng mL^−1^ using DPV in a solution of 5.0 mmol L^−1^ [Fe(CN)_6_]^3−/4−^ with 0.10 mol L^−1^ KCl (Figure 5A). The sensitivity of the immunosensor response increased from 15 to 30 min since more time was provided for the formation of the immunocomplex. The immunocomplex remained stable after 30 min. The Kd of the immunoelectrode was calculated at each time interval. The Kd values were 19.6 ± 0.9 × 10^−10^ mol L^−1^, 3.90 ± 0.05 × 10^−10^ mol L^−1^, 1.22 ± 0.02 × 10^−10^ mol L^−1^, 1.157 ± 0.006 × 10^−10^ mol L^−1^, and 1.153 ± 0.006 × 10^−10^ mol L^−1^ for incubation times of 15, 20, 25, 30, and 35 min, respectively. The lowest Kd value indicated the saturated formation of the antigen-antibody immunocomplex and was achieved in 30 min. Therefore, 30 min was the optimal incubation time for antigen-antibody immunocomplex formation.

The optimization studies showed that the best fabrication conditions for the immunosensor were an MG loading of 8.0 µg, 10 cycles of GQDs electrodeposition, a TH-monomer concentration of 0.25 mmol L^−1^, a concentration of Anti-PSA at 40 µg mL^−1^, and a 30-min incubation for antigen-antibody immunocomplex formation. These optimized parameters enhanced the sensitivity of the immunosensor for PSA detection, providing reliable and accurate measurements for PCa diagnosis.

### 3.6. Analytical Performances

#### 3.6.1. Linear Range, Limit of Detection (LOD), and Limit of Quantification (LOQ)

The linear range of the proposed immunosensor was studied with different concentrations of PSA (Figure 5B). The calibration curve displayed two linear correlations between ΔI and PSA concentration, ranging from 0.0125 to 1.0 ng mL^−1^ and 1.0 to 80.0 ng mL^−1^. The respective correlation coefficients were 0.9997 and 0.9979, respectively. The calibration curves of the current change (ΔI) and PSA concentration in the range of 0.0125 to 1.0 ng mL^−1^ and the voltammogram for different PSA concentrations obtained from 0.0125 to 1.0 ng mL^−1^ are shown in (Figure 5C,D). Two different linear relationships were obtained because the electrode surface had a constant number of antibodies. As the antigen concentration increased, the number of available active antibody sites on the electrode surface correspondingly decreased. As a result, the sensitivity in the higher antigen concentration range was reduced [39]. The LOD and LOQ were determined using the formulas 3S_a_/b and 10S_a_/b, respectively, where S_a_ represents the standard deviation of the intercept value and b represents the slope of the calibration curve [40]. Both values were calculated from the first linear range of the immunosensor. The LOD was determined to be 0.005 ng mL^−1^, while the LOQ was 0.017 ng mL^−1^.

#### 3.6.2. Operational Lifetime, Storage Stability, Reproducibility, and Selectivity of the Immunosensor

To investigate the operational lifetime of the immunosensor, one immunoelectrode was used to make 12 measurements of PSA at 0.025 ng mL^−1^. The results were highly promising, with a relative standard deviation (RSD) of 0.86%, which is significantly better than the acceptable RSD values as specified by the AOAC guidelines [41]. The immunoelectrode maintained its performance over an extended period, making it a reliable tool for PSA detection (Figure 5E).

To investigate the storage stability and shelf life of the developed immunosensor, 12 immunoelectrodes were used to measure PSA levels of 0.2, 0.4, 0.6, 0.8, and 1.0 ng mL^−1^ over a period of 3 months. The BSA/Anti-PSA/PTH/GQD/MG/SPE was prepared and stored in a refrigerator at 4 °C and tested every week. The immunoelectrode was found to retain 89.86 ± 0.09% of the initial response with an RSD of 3.7% after 3 months. According to the results, the developed immunoelectrode showed good storage stability for up to 3 months (Figure 5F).

To study the reproducibility of the proposed immunosensor, six preparations of the immunoelectrode were used to detect concentrations of PSA at 0.2, 0.4, 0.6, 0.8, and 1.0 ng mL^−1^ using DPV in a solution of 5.0 mmol L^−1^ [Fe(CN)_6_]^3−/4−^ with 0.10 mol L^−1^ KCl. The RSDs of all concentrations were between 0.14 and 0.70% (Figure S4A). Also, sensitivity was similar for all six electrodes, with an RSD of 0.08% (Figure S4B), which is within the limits of the acceptable RSA value according to AOAC standards [41]. As a result, the immunoelectrode preparation had good reproducibility.

The selectivity of the developed immunosensor was studied using interferences of 50.0 ng mL^−1^ of carcinoembryonic antigen (CEA), 50.0 U mL^−1^ of cancer antigen 125 (CA125), 50.0 U mL^−1^ of cancer antigen 153 (CA153), 50.0 U mL^−1^ of cancer antigen 199 (CA199), 0.2 mg L^−1^ of human serum albumin (HSA), 1.0% of bovine serum albumin (BSA), 1.0 mmol L^−1^ of uric acid (UA), 1.0 mmol L^−1^ of ascorbic acid (AA), and 10.0 mmol L^−1^ of glucose (Glu). These were higher concentrations than the normal concentrations found in real samples [42]. Each interference was measured by itself and all mixed together with PSA at 0.4 ng mL^−1^ and 40.0 ng mL^−1^ (Figure S5). All of the interferences had very low responses, and the changes in the current response were smaller than five percent in every single instance. No significant difference (*p* > 0.05) was observed between the PSA response and the mixtures containing interferences, confirming that the developed immunosensor provides high selectivity for PSA detection.

In these studies, the developed sensor demonstrated a wide linear range, low LOD and LOQ, excellent operational lifetime, and good storage stability. The immunosensor also exhibited good reproducibility and selectivity, confirming its potential for reliable and accurate PSA detection for PCa diagnosis.

### 3.7. Real Samples Analysis

Before quantifying PSA in human serum samples, the matrix effect was first studied in ten human serum samples. The sensitivity of calibration curves for standard PSA and human serum samples spiked with a standard (matrix-matched calibration curve) was compared using two-way ANOVA. According to the curves, there was not a significant difference in sensitivity between the standard calibration curve and the matrix-matched calibration curve at the 95% confidence level (*p* > 0.05). This indicates that there was no significant difference between the two. This means that there was no matrix effect on the response of the immunosensor. Therefore, the detection of PSA could be calculated from the calibration equation of a standard curve.

**Figure 6:**
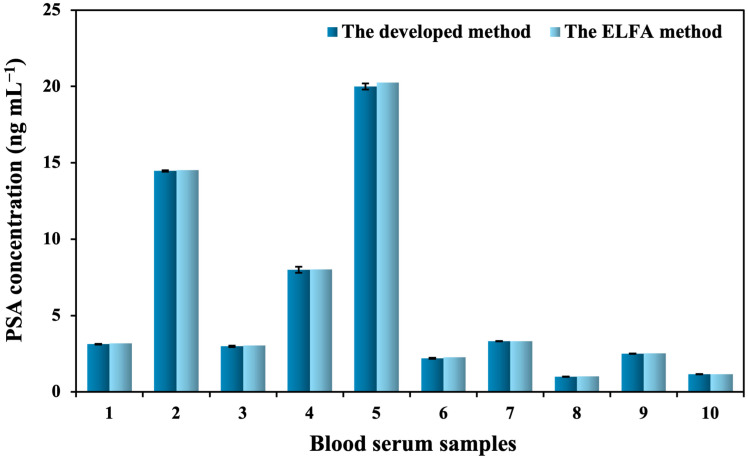
Comparison of PSA concentrations measured using the proposed PSA immunosensor (*n* = 3) and the enzyme-linked fluorescence assay (ELFA).

In order to identify the presence of PSA in serum samples, a volume of 2.0 µL of sample was carefully transferred onto the BSA/Anti-PSA/PTH/GQD/MG/SPE and then incubated for a duration of 30 min at room temperature. After 30 min, the PSA/BSA/Anti-PSA/PTH/GQD/MG/SPE was rinsed with 0.1 mol L^−1^ of PB (pH 7.4), and PSA was determined by DPV. The concentration of PSA that was determined using the developed method in ten serum samples was compared with the reference values that were obtained from the hospital using the ELFA. This comparison was carried out using a paired *t*-test. The results of the two methods were found to be in good agreement, providing a high level of confidence in the accuracy of the developed method (Figure 6).

PSA-spiked human serum was also analyzed. Human serum samples were spiked with standard PSA at 20 and 40 ng mL^−1^, and percent recoveries were calculated. Recoveries ranged from 92.4 ± 0.8 to 100 ± 2 with an RSD less than 1.86% (Table S1). The developed immunosensor therefore showed excellent reliability and accuracy.

The analysis of real human serum samples demonstrated that the developed immunosensor could accurately and reliably quantify PSA levels without significant matrix effects. The excellent agreement between the immunosensor results and the ELFA reference method, along with the high recovery rates for spiked samples, confirmed the accuracy and applicability of the developed immunosensor for detecting PSA in clinical samples.

The capabilities of the immunosensor that was developed were evaluated in comparison to those of PSA immunosensors that were previously reported (Table S2). The proposed immunosensor provided a wide linear range and a low detection limit, without the necessity for labeling or expensive components. The immunosensor showed the lowest Kd value compared to both unlabeled aptamer-based sensors [43,44,45,46,47] and unlabeled antibody-based sensors [48,49]. This low Kd value indicates the high affinity of the antigen-antibody binding, suggesting the formation of a strong and tight bond between PSA and the antibody that enhances PSA detection. Although the Kd value is slightly higher than that of a labeled antibody on APTES/biotin-streptavidin [50], the unlabeled method offers significant advantages of simplicity and cost-effectiveness. The proposed immunosensor demonstrated excellent storage stability, maintaining over 90% of its optimal performance for 12 weeks. This performance is superior to many other sensors, such as those using APTES/biotin-streptavidin and CTES-ITO-PET, which maintained stability for up to eight weeks, and HRP-Ab/AuNPs/CHI, which is stable for three weeks. In summary, the developed immunosensor stands out for its wide linear range, low detection limit, high binding affinity (low Kd value), and excellent storage stability, making it a highly effective and reliable tool for PSA detection.

## 4. Conclusions

This study presents the development of a high-performance PSA immunosensor, representing a significant advancement in prostate cancer detection. The immunosensor was fabricated and characterized using a screen-printed electrode modified with activated carbon from marigold flower (MG) and graphene quantum dots (GQD). GQDs played a crucial role in reducing the background current during PSA detection, resulting in an increased current response. The combination of MG and GQDs increased the active surface area for the polymerization of thionine, thereby enhancing the specific surface area of the electrode and increasing the number of immobilized antibodies on the immunoelectrode. The antigen-antibody dissociation constants (Kd) were calculated to be 1.157 ± 0.006 × 10^−10^ mol L^−1^, indicating a high-affinity interaction within the antigen-antibody immunocomplex. The developed immunosensor showed a linear range of 0.0125 to 1.0 ng mL^−1^ and 1.0 to 80.0 ng mL^−1^, with a detection limit of 0.005 ng mL^−1^ and a limit of quantification of 0.017 ng mL^−1^. The unlabeled electrochemical immunosensor successfully developed PSA in human serum samples, achieving recovery values ranging from 92.4 ± 0.8 to 100 ± 2 and showing excellent specificity and selectivity for clinical diagnosis. These findings highlight the immunosensor as a reliable and effective tool for accurate PSA detection, contributing to improved diagnostic capabilities for prostate cancer.

## Data Availability

The data presented in this study are available on request from the corresponding authors.

## Supplementary Materials

The following supporting information can be downloaded at: https://www.mdpi.com/article/10.3390/bios14120589/s1, Figure S1: Electrochemical characterization studies were performed in a solution of 5.0 mmol L^−1^ [Fe(CN)_6_]^3−/4−^ with 0.10 mol L^−1^ KCl at PSA 1.0 ng mL^−1^. (A) shows the cyclic voltammograms produced at each electrode modification [Bare SPE (black line), MG/SPE (orange line), GQD/MG/SPE (purple line), PTH/GQD/MG/SPE (blue line), Anti-PSA/PTH/GQD/MG/SPE (green line), BSA/Anti-PSA/PTH/GQD/MG/SPE (dark blue line), and PSA/BSA/Anti-PSA/PTH/GQD/MG/SPE (red line)]. (B) shows the EIS spectrum of each electrode modification; Figure S2: The specificity of PSA detection was performed in a solution of 5.0 mmol L^−1^ [Fe(CN)_6_]^3−/4−^ with 0.10 mol L^−1^ KCl at 1.0 ng mL^−1^ of PSA; Figure S3: The bar charts show the oxidation current density (A) and reduction current density (B) in response to various amounts of marigold flower activated carbon (MG), and the oxidation current density (C) and reduction current density (D) in response to various loadings of graphene quantum dots (GQD). The responses were measured at various GQD/MG/SPEs in a solution of 5.0 mmol L^−1^ [Fe(CN)_6_]^3−/4−^ with 0.10 mol L^−1^ KCl; Figure S4: (A,B) show the reproducibility of six electrode preparations of the PSA immunoelectrode; Figure S5: The selectivity of the developed BSA/Anti-PSA/PTH/GQD/MG/SPE was tested against various interferences separately and combined in two concentrations of PSA in a solution of 5.0 mmol L^−1^ [Fe(CN)_6_]^3−/4−^ with 0.10 mol L^−1^ KCl; Table S1: Percent recoveries of PSA, and relative standard deviations are reported; Table S2: An overview of analytical performances of recently reported PSA immunosensors. References [51,52,53,54,55,56] are cited in Supplementary Materials.
