# Supplementary material for: An Unlabeled Electrochemical Immunosensor Uses Poly(thionine) and Graphene Quantum Dot-Modified Activated Marigold Flower Carbon for Early Prostate Cancer Detection
Source: Biosensors (Basel). 2024 Dec 2;14(12):589. doi: 10.3390/bios14120589 (PMC11674062; doi:10.3390/bios14120589)
Supplement: Supplementary file 1 [file biosensors-14-00589-s001.zip › biosensors-3230019-supplementary.pdf]

biosensors

MDPI

An unlabeled electrochemical immunosensor uses poly(thionine) and graphene quantum dot-modified activated marigold flower carbon for early prostate cancer detection

Suparat Cotchim[1,2,3], Supatinee Kongkaew[1,2,3], Panote Thavarungkul[1,2,4], Proespichaya Kanatharana[1,2,4], and Warakorn Limbut[1,2,3,5,*]

S1. Electrochemical characterizations

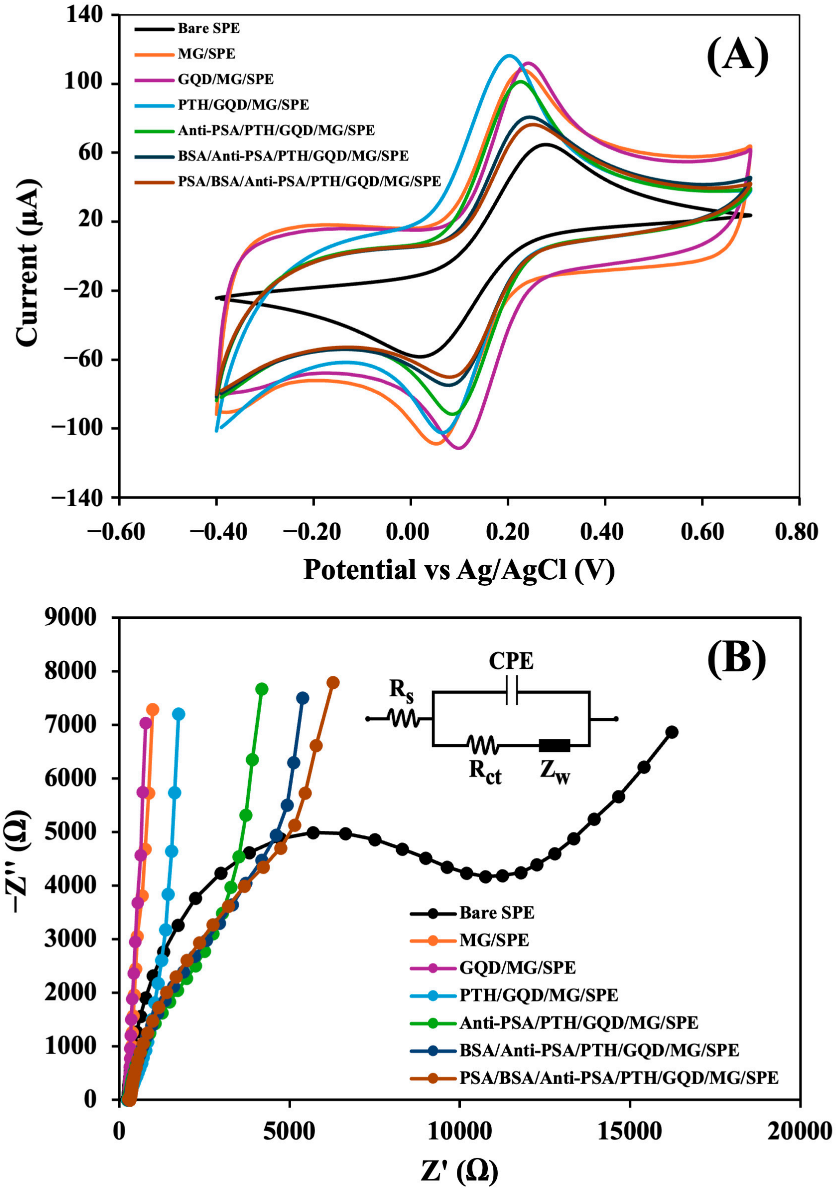

Figure S1. Electrochemical characterization studies were performed in a solution of 5.0 mmol L^{-1} $[Fe(CN)_6]^{3-/4-}$ with 0.10 mol L^{-1} KCl at PSA 1.0 ng mL^{-1}. (**A**) shows the cyclic voltammograms produced at each electrode modification [Bare SPE (black line), MG/SPE (orange line), GQD/MG/SPE (purple line), PTH/GQD/MG/SPE (blue line), Anti-PSA/PTH/GQD/MG/SPE (green line), BSA/Anti-PSA/PTH/GQD/MG/SPE (dark blue line), and PSA/BSA/Anti-PSA/PTH/GQD/MG/SPE (red line)]. (**B**) shows the EIS spectrum of each electrode modification.

S2. The specificity of PSA detection

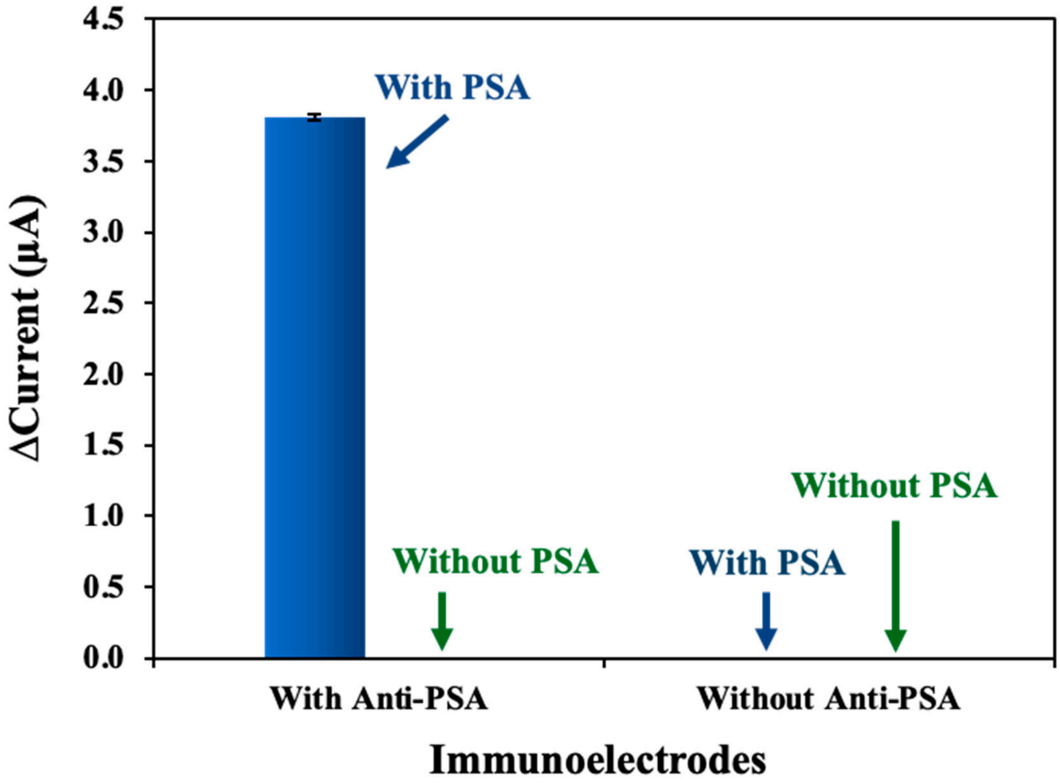

Figure S2. The specificity of PSA detection was performed in a solution of 5.0 mmol L^{-1} $[Fe(CN)_6]^{3-/4-}$ with 0.10 mol L^{-1} KCl at 1.0 ng mL^{-1} of PSA.

S3. The effect of marigold flower activated carbon (MG) and graphene quantum dots (GQD) loadings on the redox reaction at the immunosensor

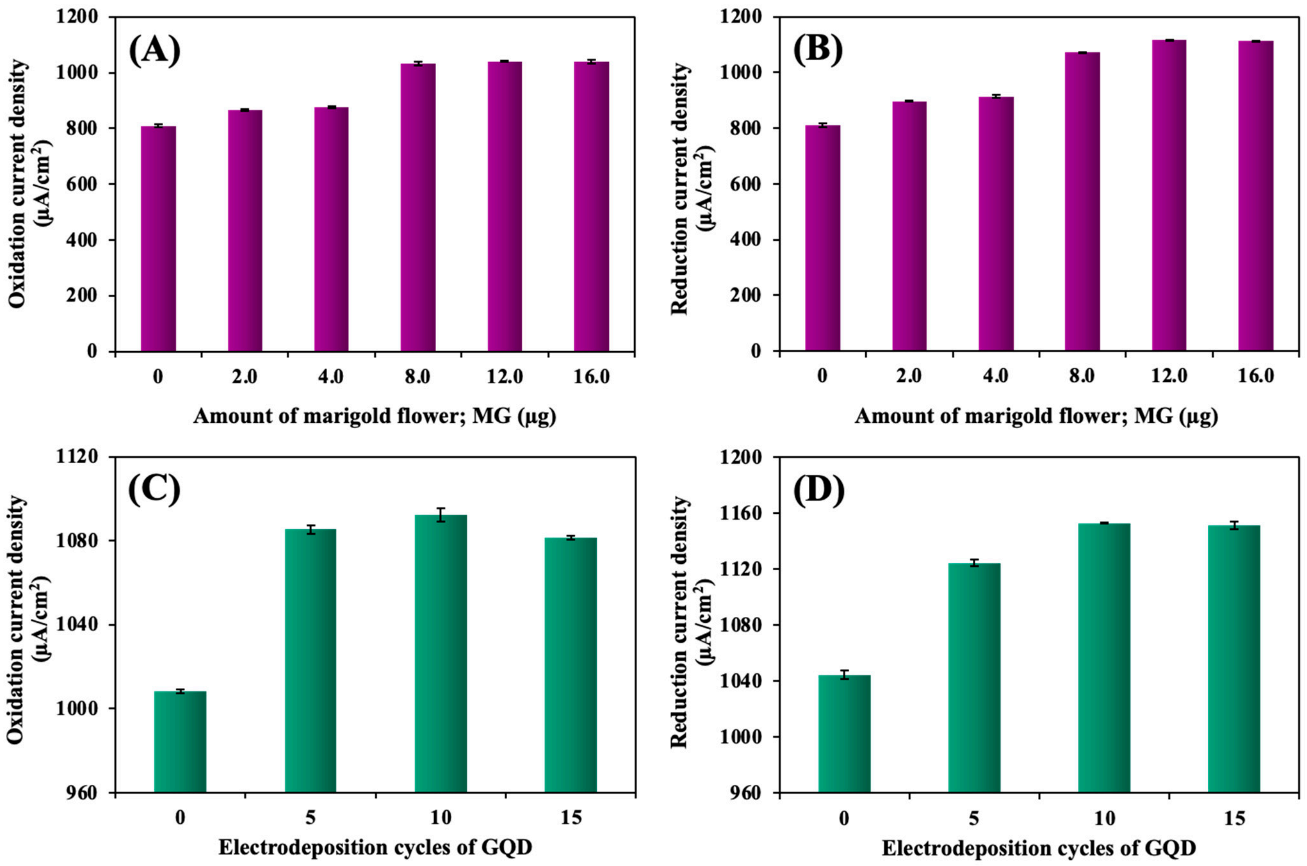

Figure S3. The bar charts show the oxidation current density (**A**) and reduction current density (**B**) in response to various amounts of marigold flower activated carbon (MG), and the oxidation current density (**C**) and reduction current density (**D**) in response to various loadings of graphene quantum dots (GQD). The responses were measured at various GQD/MG/SPEs in a solution of 5.0 mmol L^{-1} $[Fe(CN)_6]^{3-/4-}$ with 0.10 mol L^{-1} KCl.

S4. The reproducibility of the immunosensor fabrication

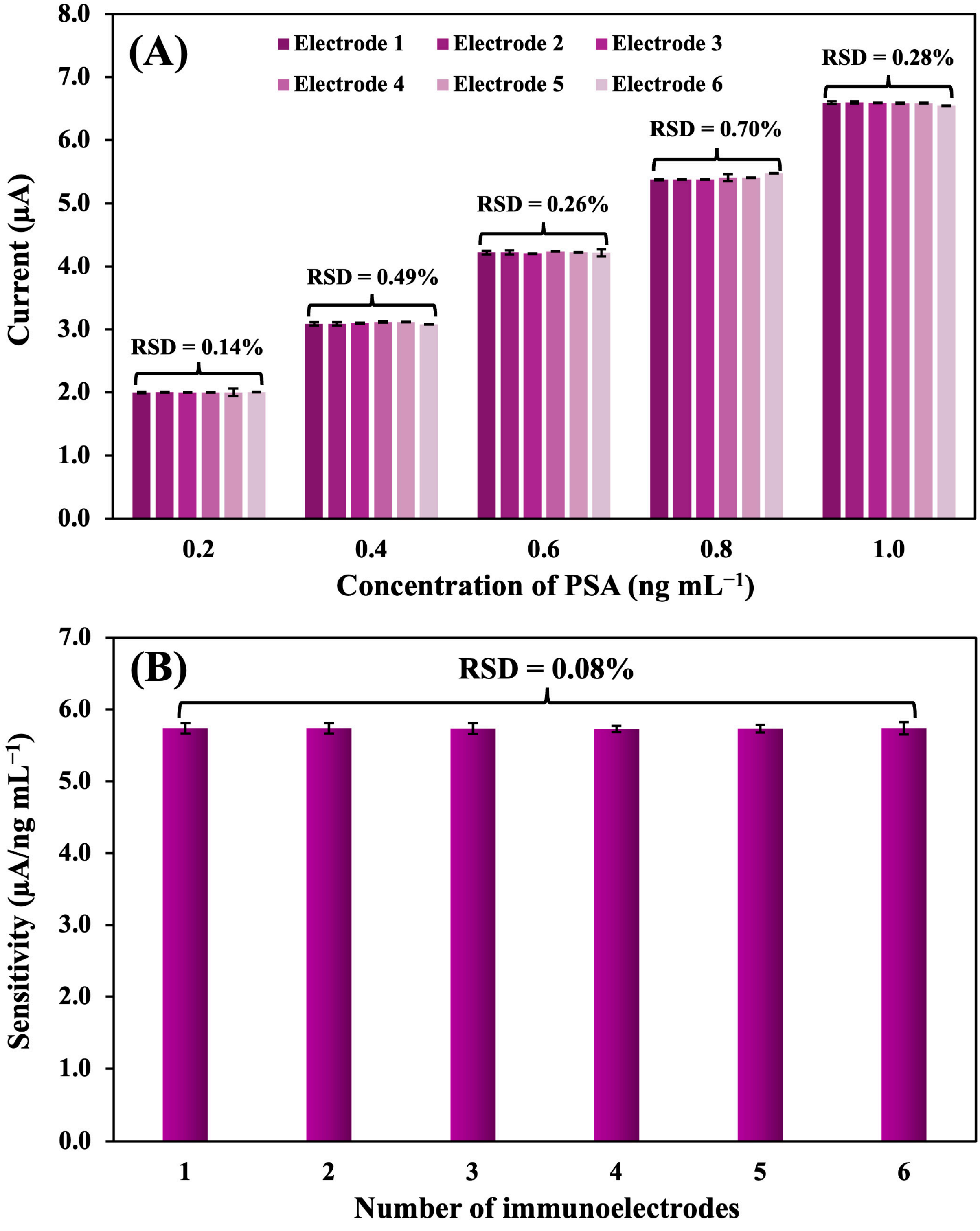

Figure S4. A and **B** show the reproducibility of six electrode preparations of the PSA immunoelectrode.

S5. The selectivity of the immunosensor

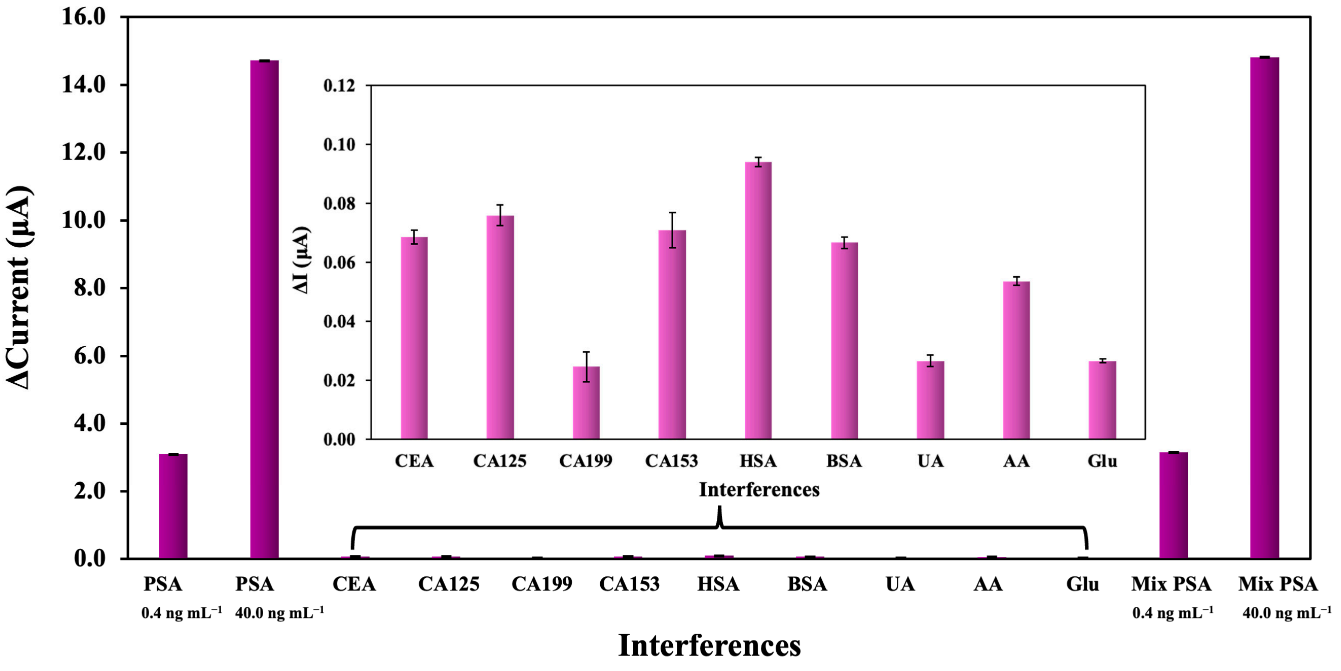

Figure S5. The selectivity of the developed BSA/Anti-PSA/PTH/GQD/MG/SPE was tested against various interferences separately and combined in two concentrations of PSA in a solution of 5.0 mmol L^{-1} $[Fe(CN)_6]^{3-/4-}$ with 0.10 mol L^{-1} KCl.

Table S1. Percent recoveries of PSA, and relative standard deviations are reported.

Sample	Concentration (ng mL^{-1})			Apparent	
	**Original**	**Spiked**	**Detected**	**Recovery (%)**	**RSD (%)**
Blood serum (#1)	3.12±0.02	20	22.0±0.2	94.3±0.9	0.78
		40	41.68±0.08	96.4±0.2	0.19
Blood serum (#2)	14.47±0.05	20	33.58±0.08	95.6±0.4	0.24
		40	53.70±0.09	98.1±0.2	0.16
Blood serum (#3)	2.98±0.04	20	21.9±0.1	94.7±0.5	0.50
		40	40.9±0.3	94.7±0.8	0.82
Blood serum (#4)	8.0±0.2	20	27.2±0.1	96.2±0.7	0.48
		40	46.3±0.1	95.8±0.3	0.23
Blood serum (#5)	20.0±0.2	20	39.3±0.2	96±1	0.60
		40	57.0±0.3	92.4±0.8	0.55
Blood serum (#6)	2.19±0.04	20	21.6±0.2	97±1	0.99
		40	41.2±0.5	98±1	1.16
Blood serum (#7)	3.316±0.004	20	22.8±0.2	97.4±0.8	0.67
		40	43.3±0.8	100±2	1.86
Blood serum (#8)	1.007±0.008	20	20.50±0.02	97.45±0.09	0.09
		40	40.1±0.2	97.7±0.4	0.40
Blood serum (#9)	2.50±0.01	20	22.11±0.02	98.1±0.1	0.10
		40	41.2±0.3	96.8±0.7	0.64
Blood serum (#10)	1.162±0.003	20	20.42±0.01	96.26±0.05	0.05
		40	40.74±0.04	98.95±0.09	0.09

Table S2. An overview of analytical performances of recently reported PSA immunosensors

**Materials**	**Type**	**Recognition elements**	**Linear range (ng mL^{-1})**	**LOD (pg mL^{-1})**	**Kd value (x10^{-10} mol L^{-1})**	**Method**	**Stability (>90%)**	**Ref**
APTES/biotin streptavidin	Labeled	Antibody	0.03-100	19	0.676	MPQ	8 weeks	[1]
HRP-Ab/AuNPs/ CHI	Labeled	Antibody	1-18	1	-	SWV	3 weeks	[2]
PANI/AuNP-PNT	Labeled	Antibody	1-100	680	-	CA	3 weeks	[3]
Pd/NH_2-ZIF-67	Labeled	Antibody	0.0001-50	0.03	-	Amp	4 weeks	[4]
H-Gr/PdNPs	Labeled	Aptamer	0.025-205	8	-	DPV	4 weeks	[5]
Graphene	Unlabeled	Aptamer	0.05-25	10	7902.7	Raman	-	[6]
Thiol terminated sulfo-betaine	Unlabeled	Aptamer	-	<1000	2.9	SPR	-	[7]
6-mercapto 1-hexanol	Unlabeled	Aptamer	-	-	370	EIS-QCM	-	[8]
Poly(JUG-co-JUGA)	Unlabeled	Aptamer	1-10000	-	26	SWV	-	[9]
CTES-ITO-PET	Unlabeled	Aptamer	0.000001-0.0015	0.00874	462.8	EIS	8 weeks	[10]
APBA/6-PICA	Unlabeled	Antibody	0.5-100	110	63.7	SWV	5 weeks	[11]
AuNRs/rGO	Unlabeled	Antibody	0.1-150	16	-	DPV	2 weeks	[12]
AuNP	Unlabeled	Antibody	1.0-8.0	550	-	DPV	10 days	[13]
MWCNT-GO-Fe_3O_4-MIP	Unlabeled	Antibody	0.01-100	5.4	1.20	EIS	4 weeks	[14]
NH_2-VMSF	Unlabeled	Antibody	0.01-1000	8.1	-	DPV	6 days	[15]
PTH/GQD/MG	Unlabeled	Antibody	0.0125-1.0 and 1.0-80.0	5.0	1.157 ±0.006	DPV	12 weeks	This work

APTES: (3-aminopropyl)triethoxysilane, MPQ: manetic particle quantification, HRP-Ab: horseradish peroxidase-tagged secondary antibody, CHI: chitosan, SWV: square wave voltammetry, PANI: polyaniline, AuNP: gold nanoparticle, PNT: peptide nanotube, CA: chronoamperometry, Pd NPs: palladium nanoparticles, NH_2-ZIF-67: amino-zeolitic imidazolate framework-67, Amp: amperometry, H-Gr/PdNPs: hemin functionalized graphene-conjugated palladium nanoparticles, DPV: differential pulse voltammetry, SPR: surface plasmon resonance (SPR), QCM: quartz crystal microbalance, poly(JUG-co-JUGA): 3-(5-hydroxy-1,4-dihydro-1,4-dioxonaphthalen-2(3)yl)propionic acid (JUGA), CTES: carboxyethylsilanetriol, ITO-PET: indium tin oxide polyethylene terephthalate film, APBA/6-PICA: aminophenylboronic/poly-indole-6-carboxylic acid, Au NRs/rGO: Au nanorods-functionalized reduced graphene oxide, MWCNT: multi-walled carbon nanotube, GO: graphene oxide, Fe_3O_4: magnetite, MIP: molecularly imprinted polymer, NH_2-VMSF: amino groups containing ordered mesoporous silica nanochannel film, PTH: poly(thionine), GQD: graphene quantum dot, MG: activated marigold flower carbon.

References

1. Orlov, A.V.; Burenin, A.G.; Skirda, A.M.; Nikitin, P.I. Kinetic Analysis of Prostate-Specific Antigen Interaction with Monoclonal Antibodies for Development of a Magnetic Immunoassay Based on Nontransparent Fiber Structures. *Molecules* **2022**, *27*, 8077, doi:http://dx.doi.org/10.3390/molecules27228077.
2. Suresh, L.; Brahman, P.K.; Reddy, K.R.; J.S, B. Development of an electrochemical immunosensor based on gold nanoparticles incorporated chitosan biopolymer nanocomposite film for the detection of prostate cancer using PSA as biomarker. *Enzyme and Microbial Technology* **2018**, *112*, 43-51, doi:https://doi.org/10.1016/j.enzmictec.2017.10.009.
3. Vural, T.; Yaman, Y.T.; Ozturk, S.; Abaci, S.; Denkbas, E.B. Electrochemical immunoassay for detection of prostate specific antigen based on peptide nanotube-gold nanoparticle-polyaniline immobilized pencil graphite electrode. *Journal of Colloid and Interface Science* **2018**, *510*, 318-326, doi:https://doi.org/10.1016/j.jcis.2017.09.079.
4. Dai, L.; Li, Y.; Wang, Y.; Luo, X.; Wei, D.; Feng, R.; Yan, T.; Ren, X.; Du, B.; Wei, Q. A prostate-specific antigen electrochemical immunosensor based on Pd NPs functionalized electroactive Co-MOF signal amplification strategy. *Biosensors and Bioelectronics* **2019**, *132*, 97-104, doi:https://doi.org/10.1016/j.bios.2019.02.055.
5. Zhang, G.; Liu, Z.; Fan, L.; Guo, Y. Electrochemical prostate specific antigen aptasensor based on hemin functionalized graphene-conjugated palladium nanocomposites. *Mikrochim Acta* **2018**, *185*, 159, doi:10.1007/s00604-018-2686-9.
6. Liu, S.; Huo, Y.; Bai, J.; Ning, B.; Peng, Y.; Li, S.; Han, D.; Kang, W.; Gao, Z. Rapid and sensitive detection of prostate-specific antigen via label-free frequency shift Raman of sensing graphene. *Biosensors and Bioelectronics* **2020**, *158*, 112184, doi:https://doi.org/10.1016/j.bios.2020.112184.
7. Jolly, P.; Formisano, N.; Tkáč, J.; Kasák, P.; Frost, C.G.; Estrela, P. Label-free impedimetric aptasensor with antifouling surface chemistry: A prostate specific antigen case study. *Sensors and Actuators B: Chemical* **2015**, *209*, 306-312, doi:https://doi.org/10.1016/j.snb.2014.11.083.
8. Formisano, N.; Jolly, P.; Bhalla, N.; Cromhout, M.; Flanagan, S.P.; Fogel, R.; Limson, J.L.; Estrela, P. Optimisation of an electrochemical impedance spectroscopy aptasensor by exploiting quartz crystal microbalance with dissipation signals. *Sensors and Actuators B: Chemical* **2015**, *220*, 369-375, doi:https://doi.org/10.1016/j.snb.2015.05.049.
9. Souada, M.; Piro, B.; Reisberg, S.; Anquetin, G.; Noël, V.; Pham, M.C. Label-free electrochemical detection of prostate-specific antigen based on nucleic acid aptamer. *Biosensors and Bioelectronics* **2015**, *68*, 49-54, doi:https://doi.org/10.1016/j.bios.2014.12.033.

10. Özyurt, C.; Uludağ, İ.; Sezgintürk, M.K. An ultrasensitive and disposable electrochemical aptasensor for prostate-specific antigen (PSA) detection in real serum samples. *Analytical and Bioanalytical Chemistry* **2023**, *415*, 1123-1136, doi:10.1007/s00216-022-04309-8.
11. Martínez-Rojas, F.; Castañeda, E.; Armijo, F. Conducting polymer applied in a label-free electrochemical immunosensor for the detection prostate-specific antigen using its redox response as an analytical signal. *Journal of Electroanalytical Chemistry* **2021**, *880*, 114877, doi:https://doi.org/10.1016/j.jelechem.2020.114877.
12. Chen, S.; Xu, L.; Sheng, K.; Zhou, Q.; Dong, B.; Bai, X.; Lu, G.; Song, H. A label-free electrochemical immunosensor based on facet-controlled Au nanorods/reduced graphene oxide composites for prostate specific antigen detection. *Sensors and Actuators B: Chemical* **2021**, *336*, 129748, doi:https://doi.org/10.1016/j.snb.2021.129748.
13. de Oliveira Cândido, T.C.; Pereira, A.C.; da Silva, D.N.; Ferreira, L.F.; Tarley, C.R.T. Development of a screen-printed electrochemical immunosensor modified with gold nanoparticles for prostate-specific antigen (PSA) detection. *Journal of Solid State Electrochemistry* **2024**, doi:10.1007/s10008-024-05939-x.
14. Karami, P.; Bagheri, H.; Johari-Ahar, M.; Khoshsafar, H.; Arduini, F.; Afkhami, A. Dual-modality impedimetric immunosensor for early detection of prostate-specific antigen and myoglobin markers based on antibody-molecularly imprinted polymer. *Talanta* **2019**, *202*, 111-122, doi:https://doi.org/10.1016/j.talanta.2019.04.061.
15. Yan, L.; Xu, S.; Xi, F. Disposal Immunosensor for Sensitive Electrochemical Detection of Prostate-Specific Antigen Based on Amino-Rich Nanochannels Array-Modified Patterned Indium Tin Oxide Electrode. *Nanomaterials* **2022**, *12*, 3810, doi:http://dx.doi.org/10.3390/nano12213810.
